# Phage Display of Combinatorial Peptide Libraries: Application to Antiviral Research

**DOI:** 10.3390/molecules16053499

**Published:** 2011-04-26

**Authors:** Guillaume Castel, Mohamed Chtéoui, Bernadette Heyd, Noël Tordo

**Affiliations:** Unité Postulante des Stratégies Antivirales, CNRS URA-3015, Institut Pasteur, 25 rue du Docteur Roux, 75724 Paris Cedex 15, France

**Keywords:** phage-display, antiviral, peptide

## Abstract

Given the growing number of diseases caused by emerging or endemic viruses, original strategies are urgently required: (1) for the identification of new drugs active against new viruses and (2) to deal with viral mutants in which resistance to existing antiviral molecules has been selected. In this context, antiviral peptides constitute a promising area for disease prevention and treatment. The identification and development of these inhibitory peptides require the high-throughput screening of combinatorial libraries. Phage-display is a powerful technique for selecting unique molecules with selective affinity for a specific target from highly diverse combinatorial libraries. In the last 15 years, the use of this technique for antiviral purposes and for the isolation of candidate inhibitory peptides in drug discovery has been explored. We present here a review of the use of phage display in antiviral research and drug discovery, with a discussion of optimized strategies combining the strong screening potential of this technique with complementary rational approaches for identification of the best target. By combining such approaches, it should be possible to maximize the selection of molecules with strong antiviral potential.

## 1. Introduction

Phage display technology was first used by George Smith in 1985 [1]. It took several years to appreciate the potential of this new tool, but the increasing number of publications relating to its use attests to the versatility of this technique for many applications, including epitope mapping, the isolation of high-affinity proteins, protein engineering and drug discovery. Phage display is based on the expression of the molecules of interest (peptides or proteins) as a fusion with the amino- or carboxy-terminus of a protein present on the surface of the bacteriophage [1,2]. This makes it possible to expose combinatorial protein/peptide libraries on the surface of recombinant phages. The exposed proteins/peptides are then selected by an affinity selection procedure, based on their ability to bind a specific target, such as an antibody, an enzyme, a purified receptor, a nucleic acid or other non protein molecule [2]. The selection process consists of several iterative cycles, each comprising capture, washing and elution steps, for progressive enrichment and amplification of the phage population carrying molecules with a higher affinity for the target. These molecules are then tested individually, to assess their activity or ability to perform the desired function. We focus here on the use of phage display to search for new antiviral compounds in highly diverse peptide libraries. Antibody libraries, another popular application of phage display for the selection of antibodies with high affinity/specificity, is not discussed here.

Antiviral research is an active domain of research, as there are too few molecules available for fighting viral infections and there may even be no available treatment for many neglected viral diseases. There is an urgent need to extend our antiviral arsenal, both to control diseases caused by emerging or endemic viruses and to overcome resistant mutants selected by treatment with the available antiviral molecules. Novel strategies and high-throughput technologies are required to address this need for new drug discovery. 

**Figure 1:**
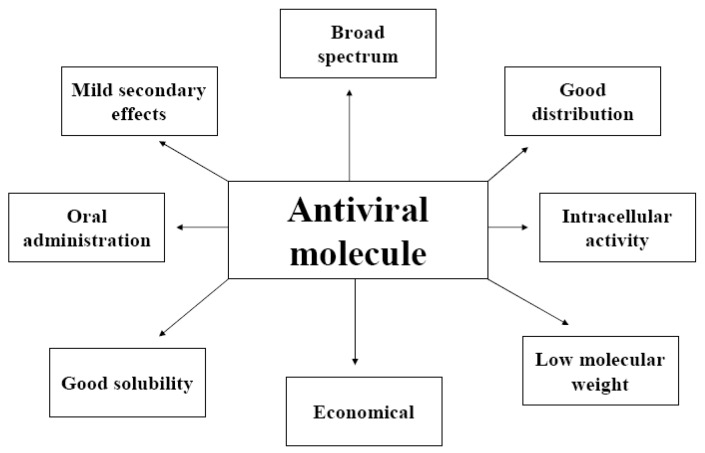
Principal characteristics of a perfect antiviral molecule.

Antiviral molecules must satisfy the same conditions as any therapeutic substance (ADME properties: absorption, distribution, metabolism, excretion): low molecular weight, good solubility, rapid elimination of the target organism, few secondary effects, simple and inexpensive production and easy administration [3] (Figure 1). Moreover, all antiviral molecules targeting the intracellular steps of the viral cycle must be able to enter cells, to express their inhibitory potential [4].

In parallel to the development of traditional molecules, such as nucleoside analogs, with well demonstrated efficacy, the pharmaceutical industry is now exploring new avenues, such as antiviral peptides. These peptides may prevent viral attachment to host cell receptors or inhibit the replication complex by interfering with protein-protein interactions, dissociating the complex and/or inhibiting its formation [4,5,6,7,8]. 

Two main approaches can be used to identify such peptides:

1. A cognitive approach based on knowledge about the structure/function of the viral components and the interactions they establish within the complex and, possibly, with cellular partners.
2. A random approach involving the high-throughput screening of highly diverse combinatorial molecule libraries generated by natural or organic chemistry (nucleic acids, peptides, proteins). This approach is particularly useful for the rapid isolation of candidate antiviral molecules [9] in cases in which little is known about the target protein, ruling out the use of more rational techniques [7].

Peptides have several advantages over traditional chemical compounds, but also a number of disadvantages. On the positive side, they are highly specific and effective, because they mimic the smallest possible part of a functional protein. They also have other very interesting therapeutic characteristics, such as high biodegradability by peptidases present in the body, limiting their accumulation in tissues and resulting in lower toxicity, because their degradation products are amino acids [3]. The potential drawbacks of peptide use are a limited ability to cross membrane barriers, a short half-life (rapid blood clearance) and potential immunogenicity [10], complex modes of action, high production costs and poor oral absorption, often necessitating intravenous administration [3,11]. Nevertheless, it is possible to overcome these limitations [3] through the use of new technologies modifying these molecules and their delivery, stability and application in preclinical settings [12]. For example, D-peptides are resistant to natural proteases (unlike L-peptides), have a longer half-life in serum, can be effectively absorbed orally [8] and are recommended for therapeutic use [13].

## 2. Isolation of Antiviral Peptides from Combinatorial Libraries by Phage Display

### 2.1. Construction of combinatorial libraries

The probability of selecting molecules with high affinity is proportional to the size of the library and, thus, to the molecular diversity of the repertoire. Combinatorial peptide libraries generally include 10^8^–10^10^ clones, but some may include as many as 10^13^ clones [14]. Generally, maximum diversity is sought [4], but fixed positions can be artificially imposed on the peptide on the basis of data obtained during a previous screening process or to reduce the range of conformations available [15].

Peptides of 6 to 45 amino acids in length may be displayed on the surface of the bacteriophage [16], but the peptide libraries generated for antiviral research usually consist of small peptides, some 6 to 15 amino acids in length (Table 1). The generation of combinatorial peptide libraries with randomized oligonucleotides consisting of NNS / NNK codons (in which N represents A, C, G or T, S represents C or G and K represents G or T) can reduce the frequency of stop codons in peptide sequences, thereby maximizing the diversity of the library [17]. Peptide structure is another key point for the maintenance of peptide functionality. The flanking sequences are particularly important as, when synthesized individually in a soluble form, many peptides totally lose the strong and specific binding affinity they displayed on the phage surface [2,18]. Thus, although linear peptides present the specific advantage of allowing many conformations and adaptability to various structures, this flexibility may decrease their affinity for the target [19]. This problem has been partly overcome by creating libraries in which the peptides are structurally constrained, to limit the number of potential conformers and to make them less dependent on the phage environment. For example, the flanking of random peptide sequences with two cysteine residues, to generate disulfide bridges, limits the number of degrees of freedom and favors certain conformations [20]. The resulting cyclic peptide has a peptide "loop" presenting the variable sequence for interaction [21]. Constrained peptides generally have a higher affinity and specificity [2,20] and a lower conformational entropy, increasing the probability that they will retain their binding capacity when removed from the phage context. In addition, the polypeptide chain must exceed a certain minimum length, to obtain a protein structurally stable in the absence of disulfide bridges [22]. For this reason, libraries of long peptides (>40 amino acids), although not exhaustive, have proved more effective for the isolation of binders than libraries of short peptides [2].

The most widely used bacteriophages are the filamentous phages infecting *E. coli,* which can grow at extremely high titers (10^12^–10^13^ pfu/mL) and have a single-strand DNA genome that is easy to manipulate [17]. It is therefore possible to present highly diverse random peptide libraries on their surface [23]. Bacteriophage M13, which tolerates the insertion of very long fragments of exogenous DNA into its genome, is an ideal tool for phage display [24,25], although other bacteriophages, such as fd, f1, T4 and T7, have also been used. Bacteriohages f1 and fd are filamentous, much like M13, and were used in pioneering studies during the development of phage display [26], whereas the use of bacteriophages T4 and T7, which have an icosahedral capsid, is much less common. Nevertheless, several studies have shown that more diverse libraries (2 to 14 times as diverse) can be presented on bacteriophage T7, with sequences displaying lower charge and hydrophobicity bias than libraries presented on bacteriophage M13 [27]. Only marginal use is made of these phages in phage display, probably because their replication induces bacterial lysis [28]. By contrast, M13 phage particles assemble in the cytoplasm and are secreted without destroying the bacteria [23], simplifying particle purification. 

For antiviral purposes, as in other applications, scientists are faced with two choices when designing combinatorial peptide libraries: which bacteriophage surface protein should be used for peptide display and what type of vector should be used for cloning? 

In theory, the peptide of interest can be fused to any surface protein, but, in practice, the pIII and pVIII are the most effective and widely used [24]. The displayed protein or peptide is usually fused to the N-terminal region, between the signal peptide and the N-terminus of the mature protein. In antiviral research, pIII is the protein of choice for the exposure of peptide libraries, because only five copies of this protein per phage are present. Moreover, pIII can accommodate large proteins and peptides with little loss of functionality concerning the ability of the phage to infect bacteria [1,29]. By comparison, the pVIII protein, although much more abundant, with 2,700 copies per phage, can display only short peptides (6 to 8 amino acids), due to geometric constraints during assembly of the phage capsid [2]. Moreover, the presence of large numbers of copies of the chimeric protein on the phage surface may favor the selection of clones expressing peptides with a lower affinity for the target (phenomenon of avidity) [25,30]. 

Three methods can be used to clone libraries of peptide genes fused to the phage coat protein gene [4,15]. The first method is based on fusion of the genes encoding the peptide or protein and the phage capsid protein in an expression vector called a phagemid [31]. A phagemid is a plasmid with the origin of replication and packaging signal of a bacteriophage and sequences for replication in *E. coli*. It is used to generate constructs encoding the phage protein fused at its N-terminus to each peptide of the library. The phagemid system requires the infection of the bacterium with a helper phage, which provides all the other necessary proteins for genome encaspidation, replication and assembly. The key advantage of a phagemid vector is that it generates one copy of each peptide per phage (monovalent display, the other proteins being encoded by the phage genome itself), preventing avidity effects due to expression of multiple copies of the peptide and therefore increasing the stringency of selection [32,33]. This system can also be used to clone large DNA fragments [19] and limits possible changes to phage infection capacity by reducing the number of chimeric proteins at the surface [31]. Indeed, after synthesis and assembly in bacteria, infectious recombinant phages are released into the culture medium, each phage containing a specific phagemid and displaying at its surface both the wild-type protein and the chimeric protein encoded by the phagemid.

The second method involves inserting the chimeric gene directly into the phage genome [4]. This is a simpler process, because infection with a helper phage is not required. However, it has the drawback that all the copies of the phage surface proteins are fused to the peptide, resulting in multivalent display and a higher risk of selecting less specific binders due to the avidity. The advantages and disadvantages of each system must be taken into account when designing libraries. Monovalent display is generally more appropriate for the characterization of high-affinity peptide inhibitors [19,32,33].

In the third method, the phage genome carries two genes, encoding two different types of protein: one recombinant and the other wild-type. The resulting phage coat is a mosaic displaying both the wild-type and the recombinant protein; it is thus possible display recombinant proteins on the phage surface even if they cannot support phage assembly [15]. 

### 2.2. Candidate selection

The affinity selection approach is based on reversible binding between a protein library (antibodies, peptides or enzymes) and a target [2]. Selected targets can be fixed onto a solid phase under conditions similar to those used in ELISA (target used to coat plastic wells) or fused to biotin captured on magnetic beads coated with streptavidin [17,25]. Several rounds of selection/amplification are required to obtain specific binders from large repertoires expressed on the surface of phages. Selection is often carried out with the purified target and involves the following steps: binding of the phages to the target, washing to remove less specific binders, elution to obtain the phages with the best affinity and, finally, the infection of fresh *E. coli* host bacteria with the selected phage for amplification [17]. The repetition of these binding-elution-amplification steps *in vitro* greatly increases the proportion of rare selected phages displaying a strong and specific interaction with the protein or peptide target in high-diversity libraries (up to 10^9^ different clones). Typically three to five rounds of selection under conditions of increasing stringency are required to isolate high-affinity binders [4].

As each phage in the library contains the nucleotide sequence encoding the peptide displayed on its surface (in its genome or in the phagemid), characterization of the selected peptides displayed on the phage is straightforward, and is based on the amplification and sequencing of the corresponding genes. When a population of recombinant phages with a highly specific binding signal is obtained, their nucleotide sequences can be analyzed to deduce the global skeleton of important amino acids, which are then fixed through the generation of a sublibrary. The inhibitory activity of the most potent selected phage sequences is then validated, either with a synthetic form of the peptides or by subcloning the corresponding gene and producing the peptide in larger amounts. Validation is based on appropriate functional antiviral assays for the virus studied, such as plaque assays [14], an immunofluorescence assays [34], inoculated cell viability assays [35] or luciferase assays [36,37].

**Figure 2:**
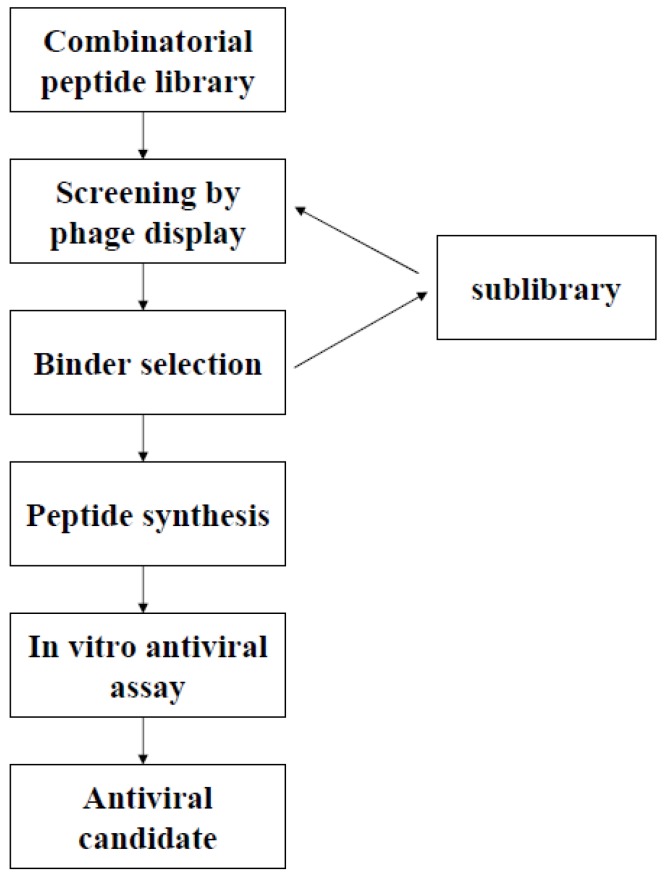
Process for the isolation of an antiviral peptide from combinatorial libraries by phage display techniques.

Phage display techniques thus have two major advantages for antiviral research. They can be used for the rapid high-throughput screening of large numbers of different molecules (up to 10^13^) in a small volume (a few microliters). The feasibility of selecting a phage with a frequency of 1/10^8^ in the original library has been demonstrated [23], for example. In addition, phage display defines a physical and molecular link between genotype and phenotype, because the gene encoding the protein (or peptide) exposed at the surface is directly sequenced and isolated from phage DNA [2]. Furthermore, some phagemid vectors can also be directly used as expression vectors, for production of the peptide/protein of interest [38]. Finally, the use of phages in a display strategy has several other specific advantages in itself: (i) the small size of the genome, simplifying the cloning steps [29]; (ii) the stability of phage particles, which can be stored at 4 °C; (iii) the efficiency of phage infection, making it possible to obtain large amounts of material, compatible with the screening of large libraries; (iv) phage display libraries can be periodically regenerated, unlike chemical libraries, which are consumed over time [2].

Traditional methods for generating new drugs involve the *in vitro* screening of hundreds of thousands of compounds against the selected targets, followed by appropriate *in vivo* activity assays. Viral diseases are a particular problem in developing countries. It must therefore be possible to produce and sell antiviral molecules as cheaply as possible [39,40]. The characteristics of phage display make this a cheap and powerful and method for discovering peptides able to bind the target with a high affinity and specificity, which could be improved to produce new therapeutic molecules [12].

However, phage display technology has several potential drawbacks that must be taken into account [2]. Clones may be toxic or may reduce phage infectivity and secretion or bacterial growth. Such clones are unlikely to be selected and are therefore likely to disappear rapidly from the library, regardless of the selection process. In addition, the level of expression of certain peptide sequences in *E. coli* is variable (depending on codon usage), introducing another potential bias into the selection process [41]. Finally, successive amplifications of the library may rapidly decrease the diversity of the library, because the clones with a growth advantage are likely to increase significantly within the population, at the expense of other clones. It has been shown that lower affinity clones may be isolated more efficiently than high-affinity clones during the selection steps in some cases [42]. This phenomenon has mostly been reported with antibody libraries, but may also operate in the case of antiviral molecules from combinatorial peptide libraries. The factors involved are not yet understood but may be related to the protein/peptide sequences displayed, affecting the efficiency of expression and/or being toxic for *E. coli*. Thus, selection may be dependent on the production of a less toxic phage-peptide and/or favored expression, rather than just the optimization of phage-peptide affinity for the target [43]. In addition, peptide motifs, such as those rich in hydrophobic residues, may interact non specifically with any molecule or surface present during experimental selection (plastic solid phase, for example) [44]. Unfortunately, as mentioned above, the selected peptides, once synthesized as soluble compounds, may not retain their optimal activity outside the microenvironment of the phage and/or in the complicated environment of a living cell or animal [25]. 

## 3. The Choice of the Target Protein: A Critical Decision

**Figure 3:**
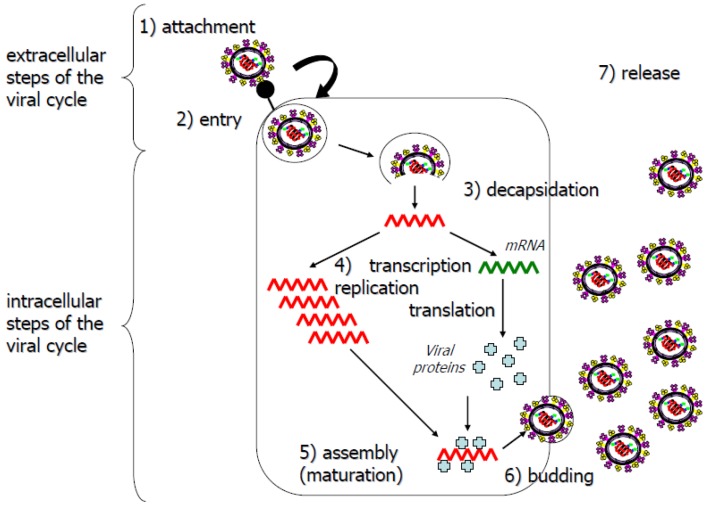
Simplified diagram of the viral life cycle. Extracellular (1, 2 and 7) and intracellular (3, 4, 5 and 6) steps are indicated.

Antiviral molecules should target key steps of the viral life cycle. These steps can be defined, in particular, by studying the structure-function relationships of viral proteins and their cellular partners. All infection steps (Figure 3) can potentially be targeted for the development of antiviral agents: penetration, release of the viral capsid or genome into the cell, transcription/replication, translation, assembly or the release of progeny virus [45]. Molecules targeting internal steps of the viral cycle are confronted by an additional challenge: entering the cell to exert their inhibitory effects. Cellular proteins essential for the viral replication cycle are also potential targets. The designers of antiviral agents tend to prefer agents with the broadest spectrum of antiviral efficiency, for reasons of both efficiency and economy [26,46]. Treatments should therefore preferentially target proteins involved in generic steps of the viral cycle [47].

The phage display of combinatorial peptide libraries has already been used successfully several times over the last 15 years, in the discovery of inhibitory peptides active against viruses (Table 1). The first viruses to be targeted were hepatitis B virus (HBV) and human immunodeficiency virus (HIV), and both these two viruses remain the most frequently targeted, but other viruses from different families were also considered. Diverse strategies and targets have been used, providing an incidental demonstration of the versatility of the method. Two main approaches are used:

(1). Targeting of the viral proteins responsible for virus entry into cells (extracellular steps)
(2). Targeting of the various elements of the viral replication complex and the cellular partners of viral proteins within infected cells (intracellular steps).

**Table 1:** Examples of antiviral peptides selected by phage display of combinatorial peptide libraries.

Viral replication step	Virus	Target	Library	Year	Ref.
Extracellular steps	IBV	whole virus	12-mer	2006	[48]
	CMV	whole virus	9-mer	1999	[49]
	Human rotavirus	whole virus	15-mer	2007	[50]
	WSSV	whole virus	10-mer	2003	[51]
	NDV	whole virus	Cyclic 7-mer	2002	[52,53]
	NDV	whole virus	7-mer	2005	[54]
	GCHV	whole virus	9-mer	2000	[55]
	ANDV	whole virus	9-mer	2009	[34]
	AIV (H9N2)	whole virus	7-mer	2009	[35,56]
	West-Nile	protein E	Mouse brain cDNA	2007	[57]
	HCV	protein E2	7-mer	2010	[59]
	HBV	protein domain PreS	12-mer	2007	[58]
	HIV-1	protein gp41	10-mer	1999	[37]
	HIV-1	protein gp41	8-mer / 7-mer	2007	[8]
	HIV-1	protein gp41	8-mer / 7-mer	2010	[13]
	Influenza A	protein HA	15-mer	2010	[14]
	SNV, HTNV, PHV	integrin alpha/beta	cyclic 9-mer	2005	[73]
Intracellular steps	HBV	protein HBcAg	6-mer	1995	[60]
	HBV	protein HBcAg	C-7-mer-C	2003	[20]
	HBV	protein HBsAg	C-7-mer-C	2005	[62]
	HIV	integrase	7-mer	2004	[65]
	HIV	protein GAG	12-mer	2005	[66]
	HIV	protein Tat	fd pVIII fragments	2005	[69]
	HIV	protein LcK	12-mer	2005	[71]
	HCV	polymerase NS5B	C-7-mer-C	2003	[63]
	HCV	polymerase NS5B	7-mer/12-mer/C-9-mer-C	2008	[64]
	HPV16	protein E2	7-mer / 12-mer	2003	[36]
	VSV	IFN receptor	7-mer	2008	[75]
	HCV	interleukin 10	15-mer	2011	[76]

### 3.1. Targeting extracellular viral replication steps

This strategy is currently the most widely used because it is easier to direct antiviral molecules, such as peptides, to extracellular targets than to direct them across the cytoplasmic (or nuclear) membrane and into the cell (or nucleus). The aim is to interfere with the attachment or internalization of the virus by blocking the interaction between the viral envelope protein and its cellular receptor. 

The most commonly used target, due to its ease of production and use, is the whole virus, purified and immobilized (assuming that it can be handled in safe conditions, correctly contained or inactivated). This method has been used for the selection of random peptides by phage display that (1) specifically inhibit cell infection and hemagglutination by the avian infectious bronchitis virus (IBV) [48]; (2) bind with high affinity to the coat protein of cucumber mosaic virus (CMV), thus providing the basis for a new antiviral strategy [49]; (3) neutralize 93% of the infectivity of the human rotavirus *in vitro* [50] and (4) prevent cell infection by the white spot syndrome of shrimp virus (WSSV) [51]. The screening of constrained cyclic heptapeptides by phage display has been used to select candidates inhibiting the hemolytic activity and spread of Newcastle disease virus (NDV) [52]. Amino acids involved in the interaction with the virus were further identified by a substitution method [53]. Three other peptides binding and partially neutralizing NDV *in vitro* were isolated in a similar manner from a different combinatorial heptapeptide library in another study [54]. Screening against the hemorrhagic carp virus (GCHV) led to the isolation of a nonapeptide giving rise to 16 variants with the potential to inhibit the virus *in vitro*. A synthetic peptide with a potentially useful inhibitory effect was designed from the sequence of the top six candidates [55]. The biopanning of a disulfide-constrained heptapeptide phage display library against AIV subtype H9N2 particles led to the selection of a phage-peptide with antiviral properties *in vitro* and *in ovo* and to the inhibition of hemagglutination activity [35]. This peptide retained its antiviral properties *in vitro* when synthesized in a linear or cyclic form. Further studies suggested that the peptide inhibited AIV replication by interacting with the HA protein and blocking its attachment to the host cell, but not its entry into cells [56]. Highly pathogenic viruses, such as the class 4 Andes virus (ANDV) have also been used as targets using in phage-based approaches (after inactivation of the purified virion by UV treatment) [34]. A cyclic 9-mer phage library was screened against ANDV, with monoclonal antibodies directed against the transmembrane surface glycoproteins Gn and Gc used to increase specificity. The most potent phage binders identified by this method inhibited the entry of ANDV into cells with a high specificity (some candidate peptides were homologous to integrin beta3, a known receptor of ANDV). For six of these binders, no significant difference in inhibition was observed between the synthetic form and the form displayed on the phage, indicating a lack of effect of the phage microenvironment on the inhibitory potential of the peptide [34].

Another approach involves the use of the purified envelope protein as a target during the selection process. This method, when applied to envelope protein E of West Nile virus (WNV), led to the selection of two peptides, of 13 and 16 amino acids, that inhibited the infection of cells with WNV and dengue virus, both of which are flaviviruses posing a major threat to public health [57]. Similarly, a peptide binding to the PreS domain of the LHBs protein of the hepatitis B virus (HBV) has been selected. The LHBs protein plays an essential role in virus infectivity and the selected peptide displayed some similarity to the LPL protein [58]. Thus, LPL is a potential receptor for HBV entry into hepatocytes and the peptide is a potential inhibitor of HBV entry. Biopanning, with the glycoprotein E2 protein of hepatitis C virus (HCV) as a target, led to the selection of a seven-amino acid peptide able to inhibit viral infectivity *in vitro* [59]. For human immunodeficiency virus type 1 (HIV-1), a “mirror-image” phage display method has been developed for the identification of D-peptides binding the hydrophobic pocket of gp41 protein and inhibiting gp41-mediated cell–cell fusion and HIV-1 infection [37]. The use of a gp41 synthesized chemically with D-amino acids as a target for the screening of a phage library expressing 10-mer peptides flanked by cysteine residues led to the identification of specific phages with L-peptide sequences. Mirror-image molecules were chemically synthesized with D-amino acids and these D-peptides bound to the natural L form of gp41. Eight of these D-peptides sharing a consensus sequence inhibited cell-cell fusion and HIV-1 entry *in vitro*, with IC_50_ values in the micromolar range [37]. These promising results led to the development of a second-generation library with constrained positions for the identified consensus residues and randomization for the other positions [8]. The screening of this constrained library against the gp41 pocket led to the selection of a family of D-peptides with improved binding properties. The accidental emergence of an 8-mer (from the 10-mer library) with apparent structural advantages resulting in stronger antiviral potency and binding led to a dedicated screening of 8-mer sequences and the isolation of a trimeric version of a peptide that was 15 times more potent as an inhibitor than the best D-peptide of the first generation. A third-generation library was then designed and screened to increase affinity for the target by optimizing flanking residues [13]. A trimeric version of a D-peptide inhibiting HIV-1 entry into cells 80 times more efficiently and with an estimated 100,000 times higher binding affinity than the best previously identified second-generation D-peptide was found.

A combinatorial phage-display pentadecapeptide (15-mer) library was recently used for multiple serial selection, with the hemagglutinin (HA) membrane proteins of subtypes H1 and H3 of influenza virus type A used as the target proteins [14]. Peptides that both bound to HA and efficiently inhibited the H1N1 and H3N2 strains of the virus *in vitro* were identified. The selected peptides resembled sialic acid, the natural cellular receptor of type A influenza virus. Secondary and tertiary selections from sublibraries increased the binding affinities of these peptides and restricted the minimum inhibitory sequence to five key amino acids.

### 3.2. Targeting intracellular viral replication steps

The aim is to select peptides binding with high affinity to replication-related proteins. Approaches of this kind are designed to inhibit the intracellular stages of viral replication, which remain a challenge in treatment, at least partly due to the lower level of accessibility of the target for the delivery of a potentially inhibitory peptide. This may account for most of the studies carried out to date being focused on viral diseases for which the commercial value of treatments is potentially high, such as those caused by HIV or the hepatitis B and C viruses. 

During HBV morphogenesis, the core protein HBcAg or the nucleocapsid interacts with the envelope protein HBsAg. A linear hexapeptide from a random peptide library selected against an HBcAg disrupted assembly with the preS domain of HBsAg, thereby inhibiting viral morphogenesis and replication [60]. The corresponding peptides were synthesized with a random mutagenesis protocol, to optimize their affinity [61]. Similar approaches have been applied to HBV, with random disulfide-constrained heptapeptide libraries. Such approaches have led to the selection of high-affinity ligands of HBcAg with stronger inhibitory effects on L-HBsAg-HBcAg association [20], or determination of the importance of specific amino-acid sequences for binding without assessing the inhibition of HBV replication [62].

For HCV, the RNA-dependent RNA polymerase (NS5B) has been used as a target for the screening, by phage display, of a disulfide-constrained heptapeptide. A peptide motif was identified that specifically inhibited NS5B activity *in vitro* by binding to the enzyme and disrupting an early step preceding the processive elongation step of RNA synthesis [63]. In another study, a peptide isolated on the basis of its affinity for the NS5B polymerase inhibited a minireplicon reconstituted in the cell cytoplasm [64]. Two-hybrid analysis and GST pulldown experiments confirmed that this peptide bound to NS5B and led to determination of the peptide binding site on NS5B while the colocalization, was evidenced by immunofluorescence analysis in infected cells. 

For obvious reasons, HIV is the virus most intensively studied. Desjobert *et al.* identified a peptide able to inhibit the strand transfer reaction catalyzed by HIV-1 integrase in a random heptapeptide phage display library, thereby opening up new prospects for treatment [65]. A 12-mer peptide binding to residues 169-191 of the capsid domain of the HIV GAG protein and inhibiting the assembly of viral particles *in vitro* was isolated from a combinatorial peptide library [66]. Variants of this peptide were synthesized to optimize affinity and *inhibition* properties. One such variant displayed strong antiviral activity in infected cells and could serve as a "lead" for the development of new anti-HIV treatments [67]. Surprisingly, when the NLS domain of the HIV Tat protein was used as a target, a peptide derived from the pVIII protein of phage fd used for screening was not only identified as a good binder [68], but also inhibited the biological functions of Tat and HIV replication in cell culture [69]. Phage display is thus a technique full of surprises, because apparently artifactual interactions can sometimes lead to the development of functional compounds! 

Outside of the “golden” HIV/hepatitis domain, very few viruses with a major impact on public health in the developed world have served as sources of targets for antiviral research based on phage display. For example, a peptide with affinity for the E2 protein of the human papillomavirus type 16 (HPV16), causing cervical cancer in women, has been shown to inhibit the transcription-altering effects of this protein on cellular genes during viral infection [36]. 

By comparison, very few attempts have been made to use phage display to find antiviral molecules active against “neglected diseases”. We are currently exploring this approach through the development of antiviral strategies for designing new drugs that will specifically interfere with the transcription/replication complex of negative-strand RNA viruses (NSRV). In humans, NSRV cause severe epidemics or even pandemics of respiratory diseases (influenza virus, respiratory syncytial virus, measles and mumps viruses), encephalitis (rabies virus, Hendra and Nipah viruses; Rift Valley Fever virus) or hemorrhagic fevers (Ebola and Marbug virus, Crimean Congo hemorrhagic fever virus, Lassa virus). Many NSRV provoke neglected diseases, several of which are assigned to biosafety level 3 or 4, because of their high levels of infectivity and the resulting death toll in humans, and some of these viruses are thought to be potential bioterrorism weapons [70]. The transcription/replication complex is an excellent target because it is similar for all NSRV: the template, consisting of the RNA genome encapsidated in the nucleoprotein (N or NP), is replicated by a virus-encoded RNA-dependent RNA polymerase complex. This should ensure both (1) specific inhibition of viral replication in the absence of cellular toxicity and (2) the selection of potentially broad-spectrum antiviral activity against many viruses, including some neglected viruses against which specific drugs are highly unlikely to be developed. Furthermore, no effective inhibitors of NSRV replicase function have yet been identified. We have begun to use this approach, with the rabies virus as a model of a neglected disease with a long incubation period (mean of 2 months), leaving a time window for the establishment of antiviral therapy, although the virus is difficult to reach once within the neuron. Consecutive panning of large libraries and then of constrained secondary libraries by phage display techniques, searching for molecules with a high affinity for the rabies virus N-RNA template, has led to the identification of five promising peptides (unpublished data). Two of these peptides also present affinity for the N-RNA template of respiratory syncytial virus (RSV) and have potentially broad-spectrum activity. This approach has been applied, in parallel, to various NSRV separated by various phylogenetic distances, with a view to identifying specific antiviral molecules with the widest possible spectrum of activity and no cellular toxicity. The best inhibitory peptides could also be used as starting points for the design of pharmacologically active peptido-mimetic molecules.

### 3.3. Targeting cellular proteins

Targeting a cellular protein playing a role in viral infection or in the antiviral immune response is a particularly appropriate approach for viruses with a high mutation frequency capable of developing resistance against drugs targeting their own proteins.

Again, much of the pioneering research in this domain was carried out on HIV, with the demonstration that a peptide isolated for its affinity for the SH3 domain of the cellular kinase Lck acts as a competitive inhibitor of the HIV Nef protein [71], opening up new perspectives for the development of anti-HIV molecules [72]. However, the use of cellular targets has the particular advantage of providing a potential response to several different viruses. For example, α_v_β_3 _integrin, which is responsible for the entry of hantaviruses into cells, has been used for the screening of a combinatorial cyclic 9-mer peptide library by phage display [73]. Seventy peptides were found to be effective competitors for this receptor and eight of these peptides had a sequence similar to a region of the viral glycoprotein. Some of the selected peptides were able to inhibit infection by Sin Nombre virus (SNV) in a focus reduction assay. In tests assessing the ability of these peptides to inhibit infection by other hantaviruses, the most promising peptides inhibited Hantaan virus (HTNV) infection more effectively than Prospect Hill virus (PHV), which uses a different receptor to enter cells. This strongly suggests that peptides act by competing with the viral glycoprotein for binding to the integrin receptor. The best four peptides, when chemically synthesized independently of the phage, retained their ability to inhibit the entry of SNV into cells, this effect being the most marked when they were used in combination [18]. 

Another strategy involves enhancing or complementing the immune response, particularly for viruses that naturally counteract innate immunity. Attempts have been made to identify compounds enhancing the immune response and capacity to resist microbial infection [74]. For example, screening of a peptide library against the IFN receptor led to the selection of two candidate peptides mimicking IFN-α antiviral activity against vesicular stomatitis virus (VSV) [75]. Interferon-α can also be stimulated indirectly, by targeting its inhibitors. A 15-mer peptide library has been screened against interleukin 10 (IL-10), a cytokine induced by the HCV core protein and involved in the poor cellular immune response against this virus. Two peptides inhibiting IL-10 were shown to restore the ability of dendritic cells to produce INF-α and to strengthen the anti-HCV T-cell response [76]. This approach is similar to the use of siRNA to target Hsp70, which is overproduced during dengue infection and favors virus multiplication by suppressing type 1 IFN production [77]. Consequently, Hsp70 is a potentially promising cellular target for a phage display strategies aiming to identify peptides active against dengue for use in treatment. 

## 4. Conclusions

Phage display is an efficient technology for selecting peptides with a high affinity for the target, but it is not always possible to obtain candidates with strong antiviral potential by this approach. However, selected peptides can subsequently be improved by rational approaches (drug design, modified residues, mutagenesis, *etc.*) [24]. In addition, phage display techniques facilitate the emergence of motifs common to selected peptides that can be used to fix positions in a secondary library, thereby retaining the best features of the selected peptides and allowing a gradual improvement in their affinity through successive selections [12]. 

We have described here success stories resulting in promising antiviral peptide candidates and demonstrating the potential of phage display for use in drug discovery. However, several similar studies have failed to select candidates with sufficient antiviral effects, even when the peptides were selected for their high affinity for the target used. On the one hand, it is not uncommon for molecules displaying significant binding when displayed on the phage surface to lose this capacity and their antiviral effect when synthesized without the carrier and tested *in vitro/in vivo*. On the other hand, a high capacity to bind the target is not necessarily associated with an antiviral effect, particularly when the binding domain is located outside the active site of the target.

A reverse strategy may therefore constitute a viable alternative approach for increasing the inhibitory potential of molecules. This would involve the initial development of functional screens [78,79], with phage display techniques then used to improve the affinity of the peptides displaying some ability to inhibit the function targeted. This rational approach would help to guide and to limit the degree of “freedom” of peptide libraries, with the phage display combinatorial approach contributing a greater diversity than would be possible with a purely rational approach [25]. The choice of amino acids constituting the candidate peptide can be left random, to provide flexibility and optimize affinity for the target [25]. Directed evolution by phage display has already been used to improve the catalytic properties of enzymes [80,81,82] and to isolate peptides mimicking interferon with stronger biological properties than the wild-type molecule [83,84]. Similarly, the characteristics of the antiviral drug palivizumab, a humanized monoclonal antibody used to treat respiratory syncytial virus (RSV) infection, have been improved by an iterative approach of mutagenesis associated with phage display [85], a method that has also proved effective for an antiviral candidate peptide [61]. Thus, although the considerable potential for the use of phage display in antiviral research is well established, it remains clear how best to use it and how (and when) to integrate it into a broader process including rational, functional and structural studies of the interactions within viral complexes and with cellular partners [24].

With a view to the development of therapeutic applications, it is important to take into account, very early in the selection strategy, all the essential parameters for the design of the molecule, which must ultimately be active in animals or humans: transmembrane penetration, metabolic stability, tissue distribution and elimination, *etc.* These data should ideally be integrated into the original library. For example, a few small peptide sequences, such as those of penetratin, Tat or VP22, are useful for promoting passage across the membrane and the specific delivery of the peptide to a cell or organ [12]. The generation of peptide libraries fused to a transmembrane signal peptide, such as the HIV Tat peptide, would make it possible to select peptides with a conformation similar to that they are likely to adopt during cell delivery. This would prevent the selection of peptides with characteristics that change during their transformation into therapeutic molecules. In any case, the use of short sequences is commercially advantageous, as it can greatly reduce the cost of production. Identification of the minimal antiviral sequence is therefore of great importance [14].

Good specificity for the target is a general requirement of the candidate peptides. However, when different strains of a virus or different viruses are targeted, extreme specificity may limit the spectrum of activity of the peptide in clinical use [14]. In this case, it is preferable to develop antiviral drugs with a broad spectrum of inhibitory activity. The choice of targets with a structure and/or function common to different viruses is the first step in the antiviral strategy [14]. Screening the library against a protein target conserved among viruses, or successive screenings of the same library against targets of similar function from different viruses could lead to the emergence of molecules with broad-spectrum potential. This approach can be completed by a functional approach involving the testing of molecules with antiviral effects against one virus against related viruses from the same family [57]. 

For a broad-spectrum approach, proteins involved in virus attachment and penetration are obvious potential targets, but elements of the viral transcription/replication complex and their cellular partners are frequently more strongly conserved and should not be ignored. As mentioned above, one target of choice is the replication ribonucleoprotein complex (RNP) of NSRV, which has a similar structure in unsegmented (Rhabdoviridae, Paramyxoviridae and Filoviridae families) and segmented (Orthomyxoviridae and Bunyaviridae families) viruses, and which typically operates independently of cellular polymerases. In addition, there is no obvious counterpart of this replication complex in humans, suggesting a low likelihood of side effects if used for treatment. Typically, the use of broad-spectrum molecules is the only way to compensate for the low economic attractiveness of neglected diseases to the pharmaceutical industry. Phage display, which has been preferentially applied to diseases with high economic potential, such as HIV or hepatitis, is a unique tool combining original strategies with an exceptional screening potential. It should be applied to neglected diseases against which no antiviral molecule is currently available. 

## References

[1] Smith G.P. (1985). Filamentous fusion phage: Novel expression vectors that display cloned antigens on the virion surface. Science.

[2] Souriau C., Hua T., Lefranc M., Weill M. (1998). Présentation à la surface de phages filamenteux: Les multiples applications du phage display. Médecine/Sciences.

[3] Decaffmeyer M., Thomas A., Brasseur R. (2008). Les médicaments peptidiques: Mythe ou réalité ?. Biotechnol. Agron. Soc..

[4] Brissette R., Goldstein N.I. (2007). The use of phage display peptide libraries for basic and translational research. Methods Mol. Biol..

[5] Loregian A., Palu G. (2005). Disruption of the interactions between the subunits of herpesvirus DNA polymerases as a novel antiviral strategy. Clin. Microbiol. Infect..

[6] Loregian A., Palu G. (2005). Disruption of protein-protein interactions: Towards new targets for chemotherapy. J. Cell Physiol..

[7] Vicent M.J., Perez-Paya E., Orzaez M. (2007). Discovery of inhibitors of protein-protein interactions from combinatorial libraries. Curr. Top. Med. Chem..

[8] Welch B.D., VanDemark A.P., Heroux A., Hill C.P., Kay M.S. (2007). Potent D-peptide inhibitors of HIV-1 entry. Proc. Natl. Acad. Sci. USA.

[9] Vidal M., Endoh H. (1999). Prospects for drug screening using the reverse two-hybrid system. Trends Biotechnol..

[10] Xie D., Yao C., Wang L., Min W., Xu J., Xiao J., Huang M., Chen B., Liu B., Li X., Jiang H. (2010). An albumin-conjugated peptide exhibits potent anti-HIV activity and long *in vivo* half-life. Antimicrob. Agents Chemother..

[11] Edwards C.M., Cohen M.A., Bloom S.R. (1999). Peptides as drugs. QJM.

[12] Ladner R.C., Sato A.K., Gorzelany J., de Souza M. (2004). Phage display-derived peptides as therapeutic alternatives to antibodies. Drug Discov. Today.

[13] Welch B.D., Francis J.N., Redman J.S., Paul S., Weinstock M.T., Reeves J.D., Lie Y.S., Whitby F.G., Eckert D.M., Hill C.P., Root M.J., Kay M.S. (2010). Design of a potent D-peptide HIV-1 entry inhibitor with a strong barrier to resistance. J. Virol..

[14] Matsubara T., Onishi A., Saito T., Shimada A., Inoue H., Taki T., Nagata K., Okahata Y., Sato T. (2010). Sialic acid-mimic peptides as hemagglutinin inhibitors for anti-influenza therapy. J. Med. Chem..

[15] Smith G.P., Petrenko V.A. (1997). Phage display. Chem. Rev..

[16] Newton J.R., Deutscher S.L. (2009). *In vivo* bacteriophage display for the discovery of novel peptide-based tumor-targeting agents. Methods Mol. Biol..

[17] Devlin J.J., Panganiban L.C., Devlin P.E. (1990). Random peptide libraries: A source of specific protein binding molecules. Science.

[18] Hall P.R., Hjelle B., Brown D.C., Ye C., Bondu-Hawkins V., Kilpatrick K.A., Larson R.S. (2008). Multivalent presentation of antihantavirus peptides on nanoparticles enhances infection blockade. Antimicrob. Agents Chemother..

[19] Lowman H.B. (1997). Bacteriophage display and discovery of peptide leads for drug development. Annu Rev. Biophys. Biomol. Struct..

[20] Ho K.L., Yusoff K., Seow H.F., Tan W.S. (2003). Selection of high affinity ligands to hepatitis B core antigen from a phage-displayed cyclic peptide library. J. Med. Virol..

[21] Ladner R.C. (1995). Constrained peptides as binding entities. Trends Biotechnol..

[22] Privalov P.L., Gill S.J. (1988). Stability of protein structure and hydrophobic interaction. Adv. Protein Chem..

[23] Sergeeva A., Kolonin M.G., Molldrem J.J., Pasqualini R., Arap W. (2006). Display technologies: Application for the discovery of drug and gene delivery agents. Adv. Drug Deliv. Rev..

[24] Pande J., Szewczyk M.M., Grover A.K. (2010). Phage display: Concept, innovations, applications and future. Biotechnol. Adv..

[25] Falciani C., Lozzi L., Pini A., Bracci L. (2005). Bioactive peptides from libraries. Chem. Biol..

[26] Scott J.K., Smith G.P. (1990). Searching for peptide ligands with an epitope library. Science.

[27] Krumpe L.R., Atkinson A.J., Smythers G.W., Kandel A., Schumacher K.M., McMahon J.B., Makowski L., Mori T. (2006). T7 lytic phage-displayed peptide libraries exhibit less sequence bias than M13 filamentous phage-displayed peptide libraries. Proteomics.

[28] Castagnoli L., Zucconi A., Quondam M., Rossi M., Vaccaro P., Panni S., Paoluzi S., Santonico E., Dente L., Cesareni G. (2001). Alternative bacteriophage display systems. Comb. Chem. High Throughput Screen..

[29] Burritt J.B., Bond C.W., Doss K.W., Jesaitis A.J. (1996). Filamentous phage display of oligopeptide libraries. Anal. Biochem..

[30] Levin A.M., Weiss G.A. (2006). Optimizing the affinity and specificity of proteins with molecular display. Mol. Biosyst..

[31] Sidhu S.S. (2001). Engineering M13 for phage display. Biomol. Eng..

[32] O'Connell D., Becerril B., Roy-Burman A., Daws M., Marks J.D. (2002). Phage *versus* phagemid libraries for generation of human monoclonal antibodies. J. Mol. Biol..

[33] Jestin J.L. (2008). Functional cloning by phage display. Biochimie.

[34] Hall P.R., Hjelle B., Njus H., Ye C., Bondu-Hawkins V., Brown D.C., Kilpatrick K.A., Larson R.S. (2009). Phage display selection of cyclic peptides that inhibit Andes virus infection. J. Virol..

[35] Rajik M., Jahanshiri F., Omar A.R., Ideris A., Hassan S.S., Yusoff K. (2009). Identification and characterisation of a novel anti-viral peptide against avian influenza virus H9N2. Virol. J..

[36] Fujii T., Austin D., Guo D., Srimatkandada S., Wang T., Kubushiro K., Masumoto N., Tsukazaki K., Nozawa S., Deisseroth A.B. (2003). Peptides inhibitory for the transcriptional regulatory function of human papillomavirus E2. Clin. Cancer Res..

[37] Eckert D.M., Malashkevich V.N., Hong L.H., Carr P.A., Kim P.S. (1999). Inhibiting HIV-1 entry: Discovery of D-peptide inhibitors that target the gp41 coiled-coil pocket. Cell.

[38] Heyd B., Pecorari F., Collinet B., Adjadj E., Desmadril M., Minard P. (2003). *In vitro* evolution of the binding specificity of neocarzinostatin, an enediyne-binding chromoprotein. Biochemistry.

[39] Knobel D.L., Cleaveland S., Coleman P.G., Fevre E.M., Meltzer M.I., Miranda M.E., Shaw A., Zinsstag J., Meslin F.X. (2005). Re-evaluating the burden of rabies in Africa and Asia. Bull World Health Organ..

[40] Massé N., Selisko B., Malet H., Peyrane F., Debarnot C., Decroly E., Benarroch D., Egloff M., Guillemot J., Alvarez K., Canard B. (2007). Le virus de la dengue: Cibles virales et antiviraux. Virologie.

[41] Rodi D.J., Soares A.S., Makowski L. (2002). Quantitative assessment of peptide sequence diversity in M13 combinatorial peptide phage display libraries. J. Mol. Biol..

[42] Malone J., Sullivan M.A. (1996). Analysis of antibody selection by phage display utilizing anti-phenobarbital antibodies. J. Mol. Recognit..

[43] Schier R., Marks J.D. (1996). Efficient *in vitro* affinity maturation of phage antibodies using BIAcore guided selections. Hum. Antibodies Hybridomas.

[44] Adey N.B., Mataragnon A.H., Rider J.E., Carter J.M., Kay B.K. (1995). Characterization of phage that bind plastic from phage-displayed random peptide libraries. Gene.

[45] Leyssen P., De Clercq E., Neyts J. (2008). Molecular strategies to inhibit the replication of RNA viruses. Antiviral Res..

[46] Aman M.J., Kinch M.S., Warfield K., Warren T., Yunus A., Enterlein S., Stavale E., Wang P., Chang S., Tang Q., Porter K., Goldblatt M., Bavari S. (2009). Development of a broad-spectrum antiviral with activity against Ebola virus. Antiviral Res..

[47] Bray M. (2008). Highly pathogenic RNA viral infections: Challenges for antiviral research. Antiviral Res..

[48] Peng B., Chen H., Tan Y., Jin M., Guo A. (2006). Identification of one peptide which inhibited infectivity of avian infectious bronchitis virus *in vitro*. Sci. Chin. C Life Sci..

[49] Gough K.C., Cockburn W., Whitelam G.C. (1999). Selection of phage-display peptides that bind to cucumber mosaic virus coat protein. J. Virol. Methods.

[50] Yao N., Yao L.G., Zhang X.M., Guo T.L., Kan Y.C. (2007). Screening for peptides of anti-rotavirus by phage-displayed technique. Sheng Wu Gong Cheng Xue Bao.

[51] Yi G., Qian J., Wang Z., Qi Y. (2003). A phage-displayed peptide can inhibit infection by white spot syndrome virus of shrimp. J. Gen. Virol..

[52] Ramanujam P., Tan W.S., Nathan S., Yusoff K. (2002). Novel peptides that inhibit the propagation of Newcastle disease virus. Arch. Virol..

[53] Chia S.L., Tan W.S., Shaari K., Abdul Rahman N., Yusoff K., Satyanarayanajois S.D. (2006). Structural analysis of peptides that interact with Newcastle disease virus. Peptides.

[54] Ozawa M., Ohashi K., Onuma M. (2005). Identification and characterization of peptides binding to newcastle disease virus by phage display. J. Vet. Med. Sci..

[55] Wang B., Ke L.H., Jiang H., Li C.Z., Tien P. (2000). Selection of a specific peptide from a nona-peptide library for *in vitro* inhibition of grass carp hemorrhage virus replication. Virus Res..

[56] Rajik M., Omar A.R., Ideris A., Hassan S.S., Yusoff K. (2009). A novel peptide inhibits the influenza virus replication by preventing the viral attachment to the host cells. Int. J. Biol. Sci..

[57] Bai F., Town T., Pradhan D., Cox J., Ashish, Ledizet M., Anderson J.F., Flavell R.A., Krueger J.K., Koski R.A., Fikrig E. (2007). Antiviral peptides targeting the west nile virus envelope protein. J. Virol..

[58] Deng Q., Zhai J.W., Michel M.L., Zhang J., Qin J., Kong Y.Y., Zhang X.X., Budkowska A., Tiollais P., Wang Y., Xie Y.H. (2007). Identification and characterization of peptides that interact with hepatitis B virus via the putative receptor binding site. J. Virol..

[59] Hong H.W., Lee S.W., Myung H. (2010). Selection of peptides binding to HCV e2 and inhibiting viral infectivity. J. Microbiol. Biotechnol..

[60] Dyson M.R., Murray K. (1995). Selection of peptide inhibitors of interactions involved in complex protein assemblies: Association of the core and surface antigens of hepatitis B virus. Proc. Natl. Acad. Sci. USA.

[61] Bottcher B., Tsuji N., Takahashi H., Dyson M.R., Zhao S., Crowther R.A., Murray K. (1998). Peptides that block hepatitis B virus assembly: Analysis by cryomicroscopy, mutagenesis and transfection. EMBO J..

[62] Tan W.S., Tan G.H., Yusoff K., Seow H.F. (2005). A phage-displayed cyclic peptide that interacts tightly with the immunodominant region of hepatitis B surface antigen. J. Clin. Virol..

[63] Amin A., Zaccardi J., Mullen S., Olland S., Orlowski M., Feld B., Labonte P., Mak P. (2003). Identification of constrained peptides that bind to and preferentially inhibit the activity of the hepatitis C viral RNA-dependent RNA polymerase. Virology.

[64] Kim M.S., Park C., Lee J.H., Myung H. (2008). Selection and target-site mapping of peptides inhibiting HCV NS5B polymerase using phage display. J. Microbiol. Biotechnol..

[65] Desjobert C., de Soultrait V.R., Faure A., Parissi V., Litvak S., Tarrago-Litvak L., Fournier M. (2004). Identification by phage display selection of a short peptide able to inhibit only the strand transfer reaction catalyzed by human immunodeficiency virus type 1 integrase. Biochemistry.

[66] Sticht J., Humbert M., Findlow S., Bodem J., Muller B., Dietrich U., Werner J., Krausslich H.G. (2005). A peptide inhibitor of HIV-1 assembly *in vitro*. Nat. Struct. Mol. Biol..

[67] Dietz J., Koch J., Kaur A., Raja C., Stein S., Grez M., Pustowka A., Mensch S., Ferner J., Moller L., Bannert N., Tampe R., Divita G., Mely Y., Schwalbe H., Dietrich U. (2008). Inhibition of HIV-1 by a peptide ligand of the genomic RNA packaging signal Psi. Chem. Med. Chem..

[68] Enshell-Seijffers D., Smelyanski L., Gershoni J.M. (2001). The rational design of a 'type 88' genetically stable peptide display vector in the filamentous bacteriophage fd. Nucl. Acids Res..

[69] Krichevsky A., Rusnati M., Bugatti A., Waigmann E., Shohat S., Loyter A. (2005). The fd phage and a peptide derived from its p8 coat protein interact with the HIV-1 Tat-NLS and inhibit its biological functions. Antiviral Res..

[70] Moran G.J., Talan D.A., Abrahamian F.M. (2008). Biological terrorism. Infect Dis. Clin. North Am..

[71] Tran T., Hoffmann S., Wiesehan K., Jonas E., Luge C., Aladag A., Willbold D. (2005). Insights into human Lck SH3 domain binding specificity: Different binding modes of artificial and native ligands. Biochemistry.

[72] Stangler T., Tran T., Hoffmann S., Schmidt H., Jonas E., Willbold D. (2007). Competitive displacement of full-length HIV-1 Nef from the Hck SH3 domain by a high-affinity artificial peptide. Biol. Chem..

[73] Larson R.S., Brown D.C., Ye C., Hjelle B. (2005). Peptide antagonists that inhibit Sin Nombre virus and hantaan virus entry through the beta3-integrin receptor. J. Virol..

[74] Tzianabos A.O. (2000). Polysaccharide immunomodulators as therapeutic agents: Structural aspects and biologic function. Clin. Microbiol. Rev..

[75] Zhang Q., Bai G., Chen J.Q., Tian W., Cao Y., Pan P.W., Wang C. (2008). Identification of antiviral mimetic peptides with interferon alpha-2b-like activity from a random peptide library using a novel functional biopanning method. Acta Pharmacol. Sin..

[76] Diaz-Valdes N., Manterola L., Belsue V., Riezu-Boj J.I., Larrea E., Echeverria I., Llopiz D., Lopez-Sagaseta J., Lerat H., Pawlotsky J.M., Prieto J., Lasarte J.J., Borras-Cuesta F., Sarobe P. (2011). Improved dendritic cell-based immunization against hepatitis C virus using peptide inhibitors of interleukin 10. Hepatology.

[77] Padwad Y.S., Mishra K.P., Jain M., Chanda S., Ganju L. (2010). Dengue virus infection activates cellular chaperone Hsp70 in THP-1 cells: Downregulation of Hsp70 by siRNA revealed decreased viral replication. Viral. Immunol..

[78] Wunner W.H., Pallatroni C., Curtis P.J. (2004). Selection of genetic inhibitors of rabies virus. Arch. Virol..

[79] Pelet T., Miazza V., Mottet G., Roux L. (2005). High throughput screening assay for negative single stranded RNA virus polymerase inhibitors. J. Virol. Methods.

[80] Strobel H., Ladant D., Jestin J.L. (2003). *In vitro* selection for enzymatic activity: A model study using adenylate cyclase. J. Mol. Biol..

[81] Soumillion P., Jespers L., Bouchet M., Marchand-Brynaert J., Winter G., Fastrez J. (1994). Selection of beta-lactamase on filamentous bacteriophage by catalytic activity. J. Mol. Biol..

[82] Pedersen H., Holder S., Sutherlin D.P., Schwitter U., King D.S., Schultz P.G. (1998). A method for directed evolution and functional cloning of enzymes. Proc. Natl. Acad. Sci. USA.

[83] Yamamoto K., Taniai M., Torigoe K., Yamamoto S., Arai N., Suemoto Y., Yoshida K., Okura T., Mori T., Fujioka N., Tanimoto T., Miyata M., Ariyasu H., Ushio C., Fujii M., Ariyasu T., Ikeda M., Ohta T., Kurimoto M., Fukuda S. (2009). Creation of interferon-alpha8 mutants with amino acid substitutions against interferon-alpha receptor-2 binding sites using phage display system and evaluation of their biologic properties. J. Interferon Cytokine Res..

[84] Kalie E., Jaitin D.A., Abramovich R., Schreiber G. (2007). An interferon alpha2 mutant optimized by phage display for IFNAR1 binding confers specifically enhanced antitumor activities. J. Biol. Chem..

[85] Wu H., Pfarr D.S., Tang Y., An L.L., Patel N.K., Watkins J.D., Huse W.D., Kiener P.A., Young J.F. (2005). Ultra-potent antibodies against respiratory syncytial virus: Effects of binding kinetics and binding valence on viral neutralization. J. Mol. Biol..

